# Electronic Sensor Technologies in Monitoring Quality of Tea: A Review

**DOI:** 10.3390/bios12050356

**Published:** 2022-05-20

**Authors:** Seyed Mohammad Taghi Gharibzahedi, Francisco J. Barba, Jianjun Zhou, Min Wang, Zeynep Altintas

**Affiliations:** 1 Institute of Chemistry, Faculty of Natural Sciences and Maths, Technical University of Berlin, Straße des 17. Juni 124, 10623 Berlin, Germany; smg@tf.uni-kiel.de; 2 Institute of Materials Science, Faculty of Engineering, Kiel University, 24143 Kiel, Germany; 3 Nutrition and Food Science Area, Preventive Medicine and Public Health, Food Sciences, Toxicology and Forensic Medicine Department, Faculty of Pharmacy, University of Valencia, 46100 Valencia, Spain; francisco.barba@uv.es (F.J.B.); jianz@alumni.uv.es (J.Z.); minwang@alumni.uv.es (M.W.)

**Keywords:** tea, polyphenol, sensor array, electronic nose, taste sensor, classifier system

## Abstract

Tea, after water, is the most frequently consumed beverage in the world. The fermentation of tea leaves has a pivotal role in its quality and is usually monitored using the laboratory analytical instruments and olfactory perception of tea tasters. Developing electronic sensing platforms (ESPs), in terms of an electronic nose (e-nose), electronic tongue (e-tongue), and electronic eye (e-eye) equipped with progressive data processing algorithms, not only can accurately accelerate the consumer-based sensory quality assessment of tea, but also can define new standards for this bioactive product, to meet worldwide market demand. Using the complex data sets from electronic signals integrated with multivariate statistics can, thus, contribute to quality prediction and discrimination. The latest achievements and available solutions, to solve future problems and for easy and accurate real-time analysis of the sensory-chemical properties of tea and its products, are reviewed using bio-mimicking ESPs. These advanced sensing technologies, which measure the aroma, taste, and color profiles and input the data into mathematical classification algorithms, can discriminate different teas based on their price, geographical origins, harvest, fermentation, storage times, quality grades, and adulteration ratio. Although voltammetric and fluorescent sensor arrays are emerging for designing e-tongue systems, potentiometric electrodes are more often employed to monitor the taste profiles of tea. The use of a feature-level fusion strategy can significantly improve the efficiency and accuracy of prediction models, accompanied by the pattern recognition associations between the sensory properties and biochemical profiles of tea.

## 1. Introduction

Tea, as one of the most popular beverages, is consumed by hundreds of millions of people around the world, due to its pleasant flavor and healthcare effects. The tea industry’s importance in the world is very high, and such that the United Nations has designated May 21 as “International Tea Day” [1]. This non-alcoholic beverage is produced from the young shoots of the tea plant (*Camellia* spp., var. *sinensis*, and var. *assamica*) [2]. Tea is cultivated in over 35 countries around the world. Most tea producing countries are in Asia (e.g., China, India, Indonesia, Sri Lanka, Iran, Japan, Bangladesh, Taiwan, Thailand, Malawi, Malaysia, etc.) and Africa (e.g., Kenya, Tanzania, Uganda, etc.), while the largest importing countries of this strategic crop are the UK, the USA, Russia, Pakistan, and Egypt [3,4]. Based on the data released by the Food and Agriculture Organization (FAO), the total global production of tea in 2018 was 8.96 million tons [5]. Although the global market value of tea during 2018–2019 increased from USD 200.105 to 215.148 billion [6], the continuation of this forward movement depends on the identification of suitable species and good practices for sustainable tea production [7].

The history of medical consumption of tea dates back to 5000 years ago in China, where tea was used to improve the immune system, blood, and urine flow, as well as to reduce joint pains [8,9]. Polyphenolic components are the main ones responsible for the antioxidant activities of this safe beverage, to quench free radicals or to form complexes with metals [10]. The promising effects of black tea on human cognitive functions probably are because of the neuroactive agents such as dopamine, kynurenic acid, and gamma-aminobutyric acid (GABA), in conjunction with phenolics [11]. Williams et al. [12] also determined this health effect in green tea, due to the presence of the main component of amino acids, namely *L*-theanine. Ma et al. [13] recently found that black tea supplementation for over seven days can considerably improve cardiovascular health in male adults, by reducing their blood pressure levels. On the other hand, Luk et al. [9] claimed that green tea, rather than black tea, has a better potential to induce antioxidant and anti-inflammatory effects, and can lessen pain progression and physical dysfunction for prevention of the progression of aging-related joint and skeletal muscle diseases such as osteoarthritis and sarcopenia. They explained that this functionality is related to the presence of bioactives, such as caffeic acid, coumaric acid, gallic acid, quinic acid, catechins, kaempferol, myricetin, quercetin, caffeine, and rutin. According to evidence-based clinical outcomes, Abe and Inoue [14] concluded that the consumption of green tea can notably reduce the risk of cardiovascular diseases (e.g., stroke), and cancer types (e.g., endometrial, esophageal, lung, non-Hodgkin’s lymphoma, oral, and ovarian).

**Figure 1:**
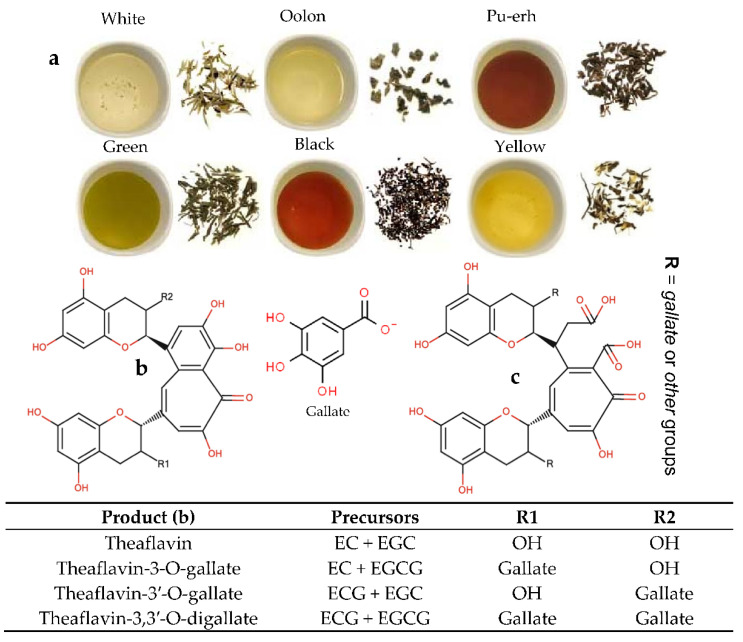
Images of different tea types (**a**) and chemical structures of theaflavins (**b**) and thearubigins (**c**) present in black tea. EC: epicatechin, EGC: epigallocatechin, ECG: epicatechin gallate, EGCG: epigallocatechin gallate.

There are six major steps in the final tea production, including harvesting, withering, leaf distortion/rolling, fermentation, firing, grading, and sorting [15]. However, depending on the fermentation degree with the oxidation of catechins (flavan-3-ols), there are several types of tea, such as green and white (un-oxidized or non-fermented), black (oxidized or fully-fermented), Pu-erh (post-fermented black tea by a microbial process), oolong (semi-fermented black tea), and yellow (similar to green tea, with additional encasing and steaming steps) (Figure 1) [16]. Among the mentioned processing steps, fermentation has a key role in tea quality, because it can significantly change the content of bioactive compounds, such as catechins and flavonoid glycosides [17]. Not only tea is rich in epicatechin (EC), epicatechin gallate (ECG), epigallocatechin (EGC), and epigallocatechin gallate (EGCG), but it also contains four epimers derived from the major catechins, including catechin (C), catechin gallate (CG), gallocatechin (GC), and gallocatechin gallate (GCG) [18]. For example, the content of the main catechins in black tea (~78%) can be reduced during fermentation through enzymatic oxidation mechanisms [19]. In general, these antioxidant agents are responsible for the bitter and astringent taste of tea and its healthy-functional characteristics [16,18]. In recent years, new groups of catechin derivatives from tea have been identified, such as phenylpropanoidated catechins and flavolignans, 6-C or 8-C-dehydroascorbic acid-EGCG; flavoalkaloids, namely 6-C or 8-C-*N*-ethyl-2-pyrrolidinone-substituted catechins; and its mixture with substitution groups of *N*-ethyl-2-pyrrolidinone and cinnamoyl in the catechin structure [20,21,22,23]. Since the content of flavoalkaloids is a function of variety, processing type, and heating intensity, these bioactives can be molecular markers to discriminate different types of tea [24]. Moreover, there are about 80 aromatic compounds in fresh leaves of tea. The count of flavoring agents can be meaningfully increased in green (>260) and black (>400) teas [25]. However, only a low number of the complex aromatic molecules formed in enzymatic (hydrolysis of glycosides by glycosidases and de novo synthesis) and nonenzymatic reactions play a significant role in their sensory perception [25,26].

Recently, analytical techniques such as spectroscopy, machine vision systems, and hyperspectral imaging have been substituted with conventional techniques to monitor chemical compounds involved in the taste, odor, and color of tea and its products (e.g., chromatographic-based techniques of high-performance liquid chromatography (HPLC) and gas chromatography-mass spectrometry (GC-MS)). These methods are very sensitive and selective; however, they are not only expensive, but also require several pretreatment steps for sample preparation. Therefore, it is essential to employ valid and fast alternatives in modern tea industries [27]. The single and combined use of an electronic nose (e-nose), electronic tongue (e-tongue), or electronic eye (e-eye) compared to conventional methods can promote the quality assessment of tea [28]. To the best of our knowledge, there are few reviews on the application of electronic sensing platforms (ESPs) for monitoring the chemical-based organoleptic attributes of tea and its products. As an update, this overview highlights the latest advancements and critical assessment of the state-of-the-art in the field of ESPs usage, to monitor the quality parameters of tea in the industry.

## 2. Non-Sensing Techniques for the Quality Assessment of Tea

Overall, the organoleptic assessment by trained test panels is a common method to evaluate the quality factors of tea type in commercial markets [29]. As mentioned, the quality sensory attributes (e.g., liquor color, flavor, bitterness/astringency, strength) are highly linked with biochemical ingredients, which can be analyzed using spectrophotometer, HPLC, and GC/GC-MS systems. For instance, theaflavins and thearubigins are responsible for color and brightness (Figure 1), while the strength of liquor tea can be a result of the quantities of caffeine and amino acids. All these chemicals can be quantified using spectrophotometer and HPLC systems [30].

Peres et al. [31] previously applied reduced flow micellar electrokinetic chromatography to determine catechins in green tea. GC/GC-MS instruments can be used to evaluate the content of the terpenoid/non-terpenoid aroma profile of various teas, according to their geographical origins [32]. Nonetheless, the high cost of these analytical systems does not allow them to be set up in tea factories. Likewise, since there is always an oxidation process during transferring the sample from the factory to the laboratory, these instruments have restrictions in adapting to routine quality inspection in a production line [33].

In addition, odor activity values, from determining aroma-active compounds, are also measured using the GC-olfactometry method. Moreover, gravimetric techniques are used to analyze the proximate composition, in terms of moisture, soluble solids, fiber, and lipid contents [34]. Koch et al. [35] used liquid chromatography-electrospray ionization-quadrupole-time-of-flight-mass spectrometry (LC-ESI-Q-TOF-MS) and atomic absorption spectrometry (flame and electrothermal) systems, respectively, to analyze the catechins-related antioxidant activity and metal content of green tea infusions.

In recent years, near-infrared spectroscopy (NIRS) with electromagnetic waves in the wavelength range of 780–2526 nm has been utilized to classify special-grade teas by quantifying indicative bioactive compounds. This mechanism is based on the response of the hydrogen-containing groups (e.g., C-H, N-H, and O-H) to NIR light. Accordingly, NIRS can be a beneficial tool for predicting tea polyphenols, catechins, theaflavins, caffeine, and chlorophyll [36,37,38]. Moreover, researchers realized that the use of NIRS or Fourier transform-NIRS (FT-NIRS) coupled chemometrics algorithms can increase the content prediction accuracy of a wide range of tea chemicals, such as phosphorus, potassium, catechins, amino acids, caffeine, theaflavins, etc. [39,40]. Dong et al. [41] found that the use of NIRS in combination with synergy interval partial least-squares regression (SI-PLS) can assess the brightness and taste intensity of a Congou black tea infusion, by predicting the ratio of theaflavins to thearubigins during fermentation. Moreover, Wang et al. [42] and Guo et al. [43] have recently evaluated the quality control of matcha tea with the accurate ratio prediction of polyphenols to amino acids using NIRS coupled with different chemometric algorithms.

## 3. Electronic Sensor Technologies: Advantages and Disadvantages in the Tea Industry

An artificial olfactory system or e-nose, similarly to chromatographic methods, can identify volatile compounds of tea using a gas sensor array. Furthermore, this technology, compared to GC-MS, is not time-consuming, high-cost, strenuous, restricted, and troublesome, and does not require complicated sample preparation [44,45]. This non-invasive, intelligent online instrument is usually coupled with a variety of materials/sensing principles, such as quartz crystal microbalance, conducting polymers, amperometry, electrochemical, surface acoustic wave, and metal oxide semiconductor (MOS; e.g., nickel, tin, cobalt, titanium, zinc, and iron oxides) sensors. Hence, the type of materials and sensors used can greatly affect the sensitivity, selectivity, efficiency, and response speed of each e-nose [28]. It was reported that the use of MOS sensors compared to the conventional method had better sensitivity to the aroma compounds of tea with long-term stability [46]. Furthermore, an e-tongue can rapidly imitate the sensory awareness of human taste by utilizing the fingerprints of response signals of tea samples [47]. These intelligent instruments are made of a sensor array to detect chemicals, accompanied by modern pattern recognition systems (PRSs). As a result, the sensor signal data can be spontaneously and authentically processed [48]. Therefore, the efficiency and speed of online quality control and inspection of tea powders and beverages using these ESPs can be significantly improved. Using an e-tongue can also prevent the accuracy loss of sensory panelists’ scores, due to their overwork and neural fatigue [29,45]. An e-eye is a bionics-based diagnostic tool for analyzing overall color and visual data. This fast technology, not only classifies different tea samples, but also determines the product quality based on their appearance-related characteristics [49].

Overall, ESPs as portable micro-instruments can assess each quality factor, without any difficult pretreatment. The same analysis using traditional detection techniques needs detailed operators and chemical reagents [27]. However, one of the disadvantages of the MOS sensor array is the high operating temperature (150–400 °C). Therefore, these devices remarkably increase consumable energy levels and require a relatively long time for heating before measuring [28]. Moreover, potentiometric sensors are the most extensively used type in e-tongues, and these platforms only respond to the charged species in solutions, limiting the count of potential analytes. Therefore, controlling the temperature and washing the electrodes are necessary to minimize the negative effects of temperature variations and the adsorption of components present in the solution on the membrane potential [27,28,29]. Moreover, another drawback of e-tongues is the relatively short lifespan of sensor materials, mainly biomaterials. Under this condition, the frequent examination of e-tongue performance is not only essential, but samples with large sizes are also often required [28,44,47]. The efficiency of e-eye systems is a function of the selection and calibration of the system components. Poor or inconsistent lighting considerably affects the acquired image quality, whereas a high-quality image provides a lower complexity and less time for image processing. Hence, assessments under this status may be more uncertain and the instrumental resolution of a signal worse. It has also been demonstrated that the performance of e-eyes can be highly influenced by processing or environmental conditions [27,28,49].

However, the achievement of precise quantitative data is needed, to find the significant associations between quality parameters and ESP signals, by employing proper algorithms [50,51]. In addition to the necessity for time-consuming trial and error for the identification of the best multivariate supervised statistical techniques, another shortcoming is the lack of optimization studies with ESPs using efficient algorithms to analyze tea quality factors [52]. This is a serious need, because a large quantity of redundant information as noise will result from the sensor array, which in turn can cause some delays due to the modeling complexity and the inadequacy of output models. Some reasons for noise formation in ESPs, such as an e-nose, include (i) the accuracy reduction of the prediction models of the mode, owing to the weakness in the response signal intensity for volatile compounds; (ii) the reduced model stability for low signal to noise ratios of e-noses under harsh conditions, such as high temperature and relative humidity; and (iii) a dissimilar sensor array with high response signal for the various volatile constituents of tea [53,54,55]. Thus, optimizing sensors toward a more stable and accurate assessment, to reduce inaccurate output information, can play an important role in better modeling quantitative data to analyze tea quality factors.

### 3.1. Electronic Nose Sensors

E-nose comprises a sensor array and a PRS. Similar to the sensory perception of human smell, the sensor array with MOS gas sensors produces a characteristic fingerprint after receiving and reacting aromatic compounds. In addition, the PRS, similarly to the human brain, can differentiate samples with diverse fingerprints [56]. Therefore, this technology, after integrating mathematical methods (Table 1), can concurrently identify volatile compounds as complex odors in the headspace of food samples such as tea. In this context, the volatile compounds present in the food aroma can be converted into electronic signals from the e-nose array, in the form of digital output. Since each volatile organic compound shows an exclusive pattern, a set of these patterns can be applied to diagnose quality changes and for discrimination purposes.

**Figure 2:**
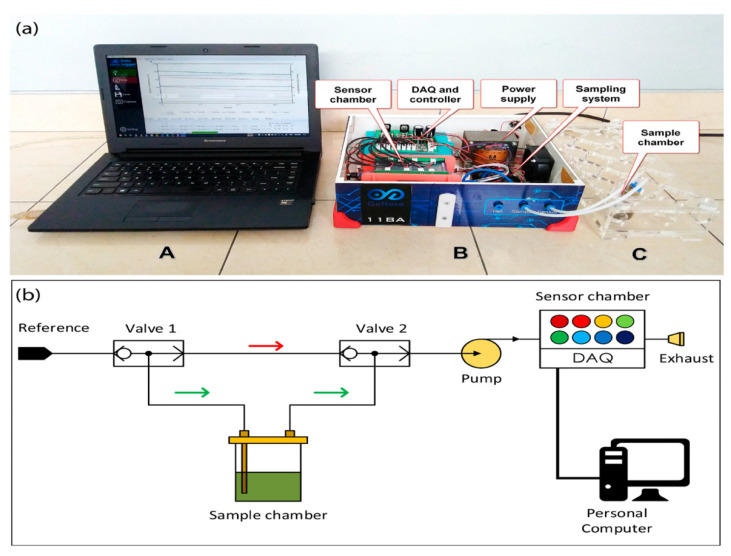
(**a**) A lab-made e-nose with eight MOS gas sensors used in the production line of a tea factory, consisting of a computer with chemometric tools (A), the main part of e-nose device equipped to a sampling system having two electronic valves (three-way system) for the airflow control (B), and the sample chamber (C), and (**b**) a graphic diagram of the e-nose tool (DAQ is the data acquisition unit). Reprinted from Hidayat et al. [57].

**Figure 3:**
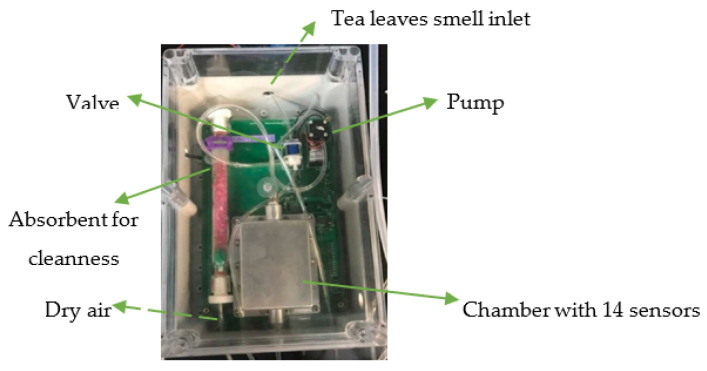
A developed gas-sensing system for smell discrimination and fermentation monitoring in the on-line production of oolong tea. The image was retrieved from Tseng et al. [58] with some modifications.

Table 1 shows a set of e-noses used to evaluate the sensory quality of various tea samples. E-noses were used to identify and discriminate different tea types, according to the origin, the optimum fermentation time, quality grades, online monitoring of bitterness and astringency levels, and adulteration degree based on the mix ratios (Table 1). Most of the gas sensors used to diagnose the tea quality were PEN 2 (10 MOS sensors), PEN 3 (10 MOS sensors), Figaro gas sensors (Osaka, Japan; 5 MOS sensors) of different models, two Alpha-MOS e-nose instruments (Fox 4000 (18 MOS sensors), and a Fox 3000 (12 MOS sensors)). Recently, Hidayat et al. (2019) designed a lab-made e-nose with eight MOS gas sensors (Taguchi, TGS, series), to assess tea quality during processing in the factory production line. These newly developed sensors, during the three stages of delay, sampling, and purging, could perform real-time analysis of tea samples at the temperature of 21 °C and relative humidity of 10% (Figure 2) [57]. Tseng et al. [58] also fabricated a new e-nose for the quality control of oolong tea. This sensor consisted of a sensor array, an adsorbent, a micro-pump with solenoid valves, and temperature and humidity sensors (Figure 3). This e-nose could be effectively used to differentiate volatile compounds and control the fermentation process in the production line of the tea factory.

**Table 1:** A summary of e-nose technologies used to monitor the quality parameters of different teas and their products.

Tea Type	e-Nose Type ^a^	Data Analysis ^b^	Utilization Purpose(s)	Keynote(s) ^b^	Reference
Indian black tea	A commercial e-nose, 4 tin oxide odor sensors	PCA, FCM, SOM, MLP, LVQ, RBF, PNN	Discriminating the flavors of various tea samples	The successful classification of teas with flavors released under different processing conditions using a RBF networked based MOS e-nose	[59]
Longjing green tea	PEN2, 10 MOS sensors	PCA, LDA, ANN	Tea grade discrimination in the industry among different cultivars	The optimum discrimination using an e-nose at 60 s, and the correct classification of 90% of the total tea samples with BPNN	[60]
13 selected tea grades	Gas sensors (Figaro Co.), 4 tin oxide sensors	MLP, RBF, CPNN	Tea quality monitoring during the tea grading process.	Tea aroma standardization in numeric terms with a classification accuracy of 90.77–93.85%	[61]
Longjing green tea	PEN2, 10 MOS sensors	PNN, BPNN, PCA, CA	The rapid quality assessment of tea grades	The identification and classification of tea quality grade using e-nose by CA and ANN	[62]
Longjing green tea	PEN2, 10 MOS sensors	PCA, LDA	Discriminating different grades of green teas	100% correct classification by LDA for five different tea samples with various qualities	[63]
Indian black tea	Gas sensors (Figaro, Japan), 5 MOS sensors	BPMLP	The quality assessment of tea via the aroma classification	Enhancing the pattern recognition accuracy of a e-nose system for black tea aroma classification	[64]
Indian black tea	Gas sensors (Figaro, Japan), 5 MOS sensors	RBF	Standardization of the e-nose tool for black tea classification	The pattern recognition algorithm for black tea aroma classification with an e-nose using a RBF neural network with the incremental learning feature	[55]
Longjing green tea	PEN2, 10 MOS sensors	PCA, LDA, BPNN	Grading the tea based on volatiles of dry tea leaf, beverages, and remains	Better discrimination of the tea grades based on their beverages using LDA and BPNN methods	[65]
Longjing green tea	PEN2,10 MOS sensors	PCA, LDA, BPNN	Recognizing the volatile components emitted by differently aged tea	Better discrimination of tea samples with leaves than their beverages and residues	[46]
Kangra orthodox black tea	Alpha M.O.S FOX 3000 EN system	SITO, MWTS	Tea classification with various fermentation times and mechanical grades	The ability improvement of an e-nose using the SITO-MWTS for online monitoring control of the tea production process	[66]
Green, Black, and Oolong teas	Odor imaging sensors array based on the reverse gel silica flat plate and the hydrophobic porous membrane	PCA, LDA	To recognize volatile organic compounds during monitoring of tea fermentation	A high potential in tea category classification with different fermentation degrees, using an e-nose based on an odor imaging sensor array	[67]
Black tea	Gas sensors (Figaro Co.), 5 MOS sensors	Bayesian	Artificial flavor perception of tea	Greater reduction in the classification error of different teas using combined sensory systems (e-nose + e-tongue) than an individual system	[68]
Xihu-Longjing green tea	Fox 4000 (Alpha MOS Co., France), 18 MOS sensors	K(PCA), K(LDA)	The quality classification of Xihu-Longjing tea	100% grade classification and recognition of tea using the KLDA-KNN model	[69]
Indian black tea	8 QCM sensors-based e-nose	-	The real-time monitoring of tea fermentation	Assessing the optimum fermentation time for 12 black tea samples with an accuracy of 96.27%	[70]
Longjing green tea	Fox 4000 (Alpha MOS Co., France), 18 MOS sensors	KLDA, KNN	Better identification of tea quality	A multi-level fusion strategy, combining e-nose and e-tongue sensors to assess tea quality	[47]
Longjing green tea	PEN3, 10 MOS sensors	KNN, SVM, MLR	Aroma compounds identification of tea	Jointly utilizing e-nose and CVS techniques to effectively identify tea quality	[44]
Longjing green tea	PEN3, 10 MOS sensors	PCA, PLSR, SVM, RF	The qualitative discrimination of tea based on volatile compounds	The best prediction of chemical components of tea using RF based on the fusion signals	[27]
Pu-erh tea	PEN3, 10 MOS sensors	CNN, PLSR, LDA	Finding a quick and accurate way to detect the type, blend ratio, and mix ratio of Pu’er tea in the industry	Higher detection ability of Pu-erh tea quality using a multi-source information fusion (e-nose and VIS/NIR spectrometer fusion)	[71]
Indian black tea	Gas sensors (Figaro, Japan), 5 MOS sensors	PCA, KNN, PLS-DA	Classifying tea samples based on aromatic compounds	Tea quality classification (accuracy = 99.75%) due to the sensitivity to different chemicals (e.g., linalool, linalool oxide, β-ionone, terpeniol, and geraniol)	[72]
Indian black tea	Gas sensors (Figaro, Japan), 5 MOS sensors	Recurrent Elman network	A rapid prediction of the optimum fermentation time of black tea	Monitoring the fermentation process of tea using an e-nose and a recurrent Elman network	[73]
Organic green teas	PEN3, 10 MOS sensors	PCA, SVM, PLSR, RF, KRR, MBPNN	The concurrent classification of tea grade and price prediction with an excellent performance	MBPNN model: able to represent the nonlinear relationship between aroma (inputs) and quality (outputs) data of tea	[74]
West Lake Longjing green tea	Self-developed e-nose system, 8 MOS sensors	CART	Quality level identification of tea types	The grading regulation of different teas based on the aroma components alone	[75]
Xihu Longjing and Pu-erh teas	MOS-based PEN3 sensors	PCA, LDA	The rapid, precise determination of the difference in the overall characteristic aromas of tea varieties	The e-nose ability to discriminate different priced Xi-hu Longjing tea samples and varying storage years of Pu-erh tea samples	[76]
Black, Green, and yellow teas	PEN3, 10 MOS sensors	Grid-SVR, XGBoost, RF	The polyphenol content in cross-category tea	Improving the estimation accuracy of tea polyphenol content for cross-category evaluation (the best model: XGBoost)	[77]
Oolong tea	MOS-based gas sensors (Figaro, USA)	-	Accurately monitoring the smell variation during fermentation, based on online tests in a tea factory	e-nose: an efficient option to replace the sensory function of panelists in the future	[58]
12 green teas	PEN3, 10 MOS sensors	SVM, CNN-Shi, CNN-SVM-Shi, CNN	A rapid, convenient, and effective method for classifying green teas from different geographical origins	High accuracy and strong strength of the CNN-SVM for the fine-grained classification of multiple highly-similar teas	[78]
Green tea (fried, baked, sunburned, and steamed)	PEN2, 15 MOS sensors	PCA, LDA, KNN	The optimization of an e-nose sensor array to identify aroma compounds of tea	Eliminating redundant sensors, improving the quality of original tea aroma data A high accuracy (94.44~100%) using combined methods of LDA and KNN	[51]
Xihu-Longjing green tea	PEN3, 10 MOS sensors	XGBoost, RF, BPNN, SVM, LightGBM	Improving the practical use of e-nose devices using TrLightGBM	TrLightGBM (transfer learning) model: the best performance for the identification of different production areas and harvest times	[79]
Longjing green tea	PEN3, 10 MOS sensors	PCA, MDS, LDA, LR, SVM	e-nose feasibility to qualitatively and quantitatively analyze quality grades of tea	A 100% accuracy for the classification of tea infusions with SVM based on the data processed by LDA	[56]
Dianhong black tea (44 infusions)	Heracles II fast GC-E-Nose (Alpha MOS Co., France)	PLS-DA, FDA	A innovative technical route for the quality evaluation and control of tea infusions	A supplement for the objective sensory assessment	[80]
Green tea (*Fudingdabai* variety)	Heracles II gas phase e-nose (Alpha M.O.S., Toulouse, France)	PCA, PLS-DA	A framework for directional processing and quality improvement of tea	High performance of a gas-phase e-nose, to quickly and effectively characterize the dynamic changes under different drying conditions of tea	[81]

^a^ MOS: metal oxide semiconductor, QCM: quartz crystal microbalance. ^b^ ANN: artificial neural networks, BP-MLP: back-propagation multilayer perceptron, BPNN: back-propagation neural network, MBPNN: multi-task framework based on BPNN, CA: cluster analysis, CART: classification and regression tree, CPNN: constructive probabilistic neural network, CNN: convolutional neural network, CVS: computer vision system, FCM: Fuzzy C-means, FDA: Fisher discriminant analysis, Grid-SVR: grid support vector regression, KNN: K-nearest neighbors, KRR: kernel ridge regression, KPCA: kernel-based PCA, KLDA: kernel-based LDA, LDA: linear discrimination analysis, LightGBM: Light gradient boosting machine, LR: Logistic regression, LVQ: learning vector quantization, MDS: multi-dimensional scaling, MLP: Multilayer perceptron, MLR: multinomial logistic regression, MWTS: moving window time slicing, PLS-DA: partial least squares discriminant analysis, PLSR: partial least squares regression, PLS-DA: partial least squares discriminant analysis, PCA: principal component analysis, PNN: probabilistic neural network, RBF: radial basis function, RF: random forest, SITO: social impact theory based optimizer, SOM: Self-organizing map, SVM: support vector machine, VIS/NIR: visible near infrared, XGBoost: extreme gradient boosting.

Currently, gas-phase e-nose (GP-e-nose) technology, along with multivariate statistical analyses (MVSA), has been utilized to determine the objective sensory evaluation of teas, with analysis of their aroma quality. Yan et al. [82] analyzed the volatile compounds of green tea using GP-e-nose and GC-ion mobility spectrometry (GC-IMS), coupled with MVSA (such as PLS-discriminant analysis (PLS-DA) and principal component analysis (PCA)). Although 33 aromatic compounds were identified, the most important aromatic ingredients were 3-methylbutanal, methyl benzoate, isopropyl alcohol, and heptanal. They also reported that the profile of volatiles was dynamically changed with an enhancement in the drying temperature. However, the GP-e-nose rapidly and successfully characterized the dynamic changes induced by high drying temperatures and complemented the findings obtained from the GC-IMS analysis. In addition, the integration of GP-e-nose and MVSA (PLS-DA and Fisher discriminant analysis (FDA)) has been used to evaluate the aroma quality of Dianhong black tea infusions [80]. They showed that aldehydes were the most frequent compounds identified among 61 volatiles. Accordingly, aromatic compounds of furan, methyl acetate, 2,3-pentanedione, limonene, and linalool had a positive correlation with the aroma quality of tea infusions, where the aroma quality was negatively influenced by the presence of 3-ethylpentane, 1-pentene, (E)-2-hexene, and methyl eugenol compounds [80]. Yan et al. [81], using an e-nose coupled with GC–MS, could analyze the major volatile aromatic components and aroma variances of black tea obtained from two tea cultivars (i.e., Fuyun 6 (mainly, sweet aroma) and Jinguanyin (mostly, floral aroma)). There were nine different aroma compounds between the two cultivars, including heptaldehyde, carveol, hexanal, furfural, 2,2,6-trimethylcyclohexanone, 2-pentadec-2-enylfuran, cis-3-hexenyl acetate, methyl salicylate, and 5-methyl-2-hexanone. The e-nose results proved that five out of the ten sensors could differentiate the two cultivars, particularly, S2 (sulfide and hydrogen sulfide), S6 (biogas, methane, and hydrocarbons), S7 (combustible gases), and S10 (combustible gases and alkanes). Recently, Song et al. [83] successfully applied an intelligent fixation as the first step in the appearance and fragrance formation of green tea. The online measurement of aromatic compounds using an e-nose and a set of other sensors coupled with fuzzy logic algorithms was performed to assess the fresh fragrance, enzymatic reaction, and charring occurrence. Moreover, Sanaeifar et al. [84], with the integration of an e-nose and confocal Raman microspectroscopy, could guarantee the tea quality, by monitoring its safety in terms of determining the pesticide residue concentration of chlorpyrifos in tea processing. Thus, it was demonstrated that the use of an e-nose coupled with chemometric strategies represented a useful tool to concurrently monitor the safety and quality of tea.

### 3.2. Electronic Tongue Sensors

**Figure 4:**
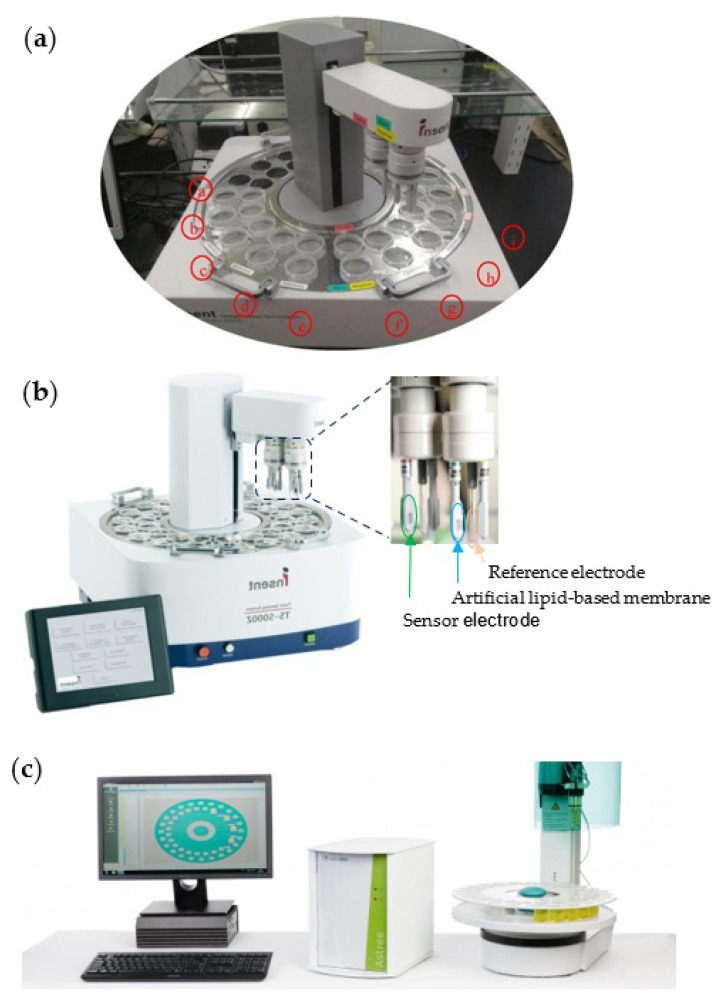
Images of three common potentiometric e-tongue systems for measuring the taste attributes of tea: (**a**) SA-402B (Intelligent Sensor Technology Co., Ltd., Japan; reprinted from Liu et al. [107]) (a, for measuring the aftertaste value; b and c, for cleaning the sample rapidly; d and e: for cleaning the positive and negative solution; f: for the positive and negative cleaning solution; g: for the sensor calibration; h: for sensor reset; i: for the liquor sample), (**b**) TS-5000Z (Insent Inc., Atsugi-Shi, Japan), and (**c**) ASTREE (Alpha MOS Inc., Toulouse, France).

An e-tongue not only can imitate the human tongue, but also can present a more sensitive, automated, and unbiased representation of tastes than human tasters. This intelligent technology comprises an array of liquid sensors to analyze liquid-based food products (such as vegetable oils) and beverages, an appropriate method of pattern recognition, and multivariate calibration for data processing. This emerging tool can be utilized after transforming gas and solid materials into their liquid forms or extracts. Therefore, the sensors can easily respond to solubilized tastes, by transducing a signal or a pattern of signals recognized by the software system [28,85]. Recent two-decade studies on the use of e-tongues in measuring the quality of tea types are summarized in Table 2. In general, the e-tongue systems were voltammetric [68,86,87,88,89,90,91,92,93,94], potentiometric [29,95], and fluorescent [45] sensor arrays. In recent years, the commercial SA-402B [96,97] and TS-5000Z [98,99,100] taste sensing systems (Insent Inc., Japan) have been applied to detect the taste quality of different tea samples (Figure 4a,b). These sensors are constructed based on Prof. Kiyoshi Toko’s idea, with different potentiometric electrodes with lipid-polymeric membranes. One of the other e-tongue systems used to analyze tea taste is the Alpha MOS ASTREE (Figure 4c) [27,47,101]. The sensing electrodes work based on the potentiometric measurement principle, to analyze the taste of liquid-based products or solids dissolved in a liquid [29]. Thus, the sensors used in e-tongue systems are mostly potentiometric to assess the taste of a broad range of teas.

Overall, the maximum number of cross-sensitive electrodes is seven, to detect tastes of umami, richness, sourness, saltiness, sweetness, bitterness, and astringency [27,95,101]. However, He et al. [29] used a potentiometric sensor array along with PCA to classify tea samples by evaluating ten organoleptic characteristics, including heavy, thick, sweet, fresh, mellow, fired, and stale flavors, as well as astringency, tenderness, and purity. The application target of e-tongue systems is the quality prediction of tea from various geographical origins and grade levels, with much better discrimination compared to the conventional methods. Yan et al. [101] showed that the integration of an e-tongue with seven independent sensors and chemometrics could efficiently diagnose the geographical origins of Anji-white tea. In addition, He et al. [29] discriminated black and green teas from various geographical origins and quality grades using an e-tongue technology based on the prediction of sensory attributes. Zhang et al. [102] recently reported the analysis of e-tongue data to classify tea according to semi-supervised learning of generative adversarial networks. The results proved that this approach could more effectively promote the accuracy of classification than a multi-class support vector machine (SVM), PLS-DA, and decision tree. Huo et al. [103], using an artificial nose and tongue with to colorimetric sensor arrays, could successfully classify green tea harvested from the same geographical origin using a hierarchical cluster analysis (HCA) algorithm.

Cheng et al. [104] assessed the use of an e-tongue and GC-MS to evaluate the effect of storage age on the metabolic profile and taste quality of a 20-year sequence of aged Qingzhuan tea. They identified 47 bioactive compounds responsible for the age variation of tea quality. They were formed through different biochemical mechanisms, such as methylation of catechins, glycosylation of flavonoids, formation of theabrownins, biosynthesis of triterpenoids, and degradation of flavoalkaloids. Although the content of polyphenols and flavonoids was enhanced at the 10th year of aging, the amount of theabrownins reached the maximum level at the 15th year of aging. Moreover, not only new triterpenoids were formed at the end of aging time, but also the quantity of initial triterpenoids over the aging time was significantly increased. Accordingly, the authors reported the improved taste of Qingzhuan tea after 10 years of storage with a decrease in bitterness and astringency values and an increase in the main quality-related bioactive compounds. Li et al. [105], using an e-tongue system and an ultrahigh performance liquid chromatograph—Orbitrap-mass spectroscopy, discriminated the taste quality and chemical profiles of 28 crush-tear-curl black tea samples, collected from six geographical regions. The results showed that teas collected from Sri Lanka, North India, China, South India, and Kenya had tastes being more umami and astringent; more umami, more sweetness, and astringent; moderate umami and sweetness; and moderate umami and astringent, respectively. The multivariate statistical analysis also revealed that teas contained 78 metabolites such as amino acids, pigments, and flavone/flavonol glycosides.

The use of e-tongues and e-noses has led to the improved performance and accuracy of tea grading and classification, using the simultaneous analysis of odor and taste. It was concluded that these combined ESPs, rather than the single systems, can significantly promote the clustering and classification rates of tea [47,68,71,90,92,103,106]. However, the classifier type for determining the best prediction model was varied (Table 2). The concurrent application of an e-tongue and e-eye based on deep learning has recently led to great achievements in rapidly discriminating Pu-erh tea stored for different times, from 0 to 8 years [92]. Zhi et al. [47] earlier realized that a synergy of modeling feature fusion (fuse the time-domain and frequency-domain based features) and decision fusion (D-S evidence to associate the classification findings from multiple classifiers) of an e-nose and e-tongue could meaningfully improve the accuracy rate for quality prediction of teas with four different grades.

**Table 2:** The e-tongues used to analyze the sensory quality factors in different types of tea.

Tea Type	e-Tongue Type	Data Analysis ^a^	Utilization Purpose(s)	Special Note(s) ^a^	Reference
Green and black teas	Voltammetric e-tongue system, 3 noble metal-type electrodes, an Ag/AgCl reference electrode, a stainless steel counter electrode	PCA	Accurately discrimination of tea samples	Classification of tea samples based on the taste attributes detected by an online e-tongue system	[86]
Green, black, and oolong teas	Voltammetric e-tongue (SA402 Anritsu Corp., Japan), 8 different lipid/polymer membranes, an Ag/AgCl reference electrode	PCA	The potential of combined sensors to detect taste attributes of tea samples	Improving the taste quality of tea samples by integrating a voltammetric e-tongue, and a potentiometric multichannel lipid membrane taste sensor	[87]
Chinese green tea	Potentiometric all-solid-state e-tongue (Alpha M.O.S. Co., France), 7 sensors (ZZ, BA, BB, CA, GA, HA, and JB)	ANN, KNN	The online grading of tea	Using e-tongue technology with ANN pattern recognition to identify tea grade level	[95]
Chinese green and black teas	Potentiometric all-solid-state e-tongue (Alpha M.O.S. Co., France), seven liquid cross-selective sensors, a reference electrode	PCA	A rapid test for diagnosing taste quality of tea samples	Predicting sensory characteristics and their relationship to the taste quality of tea assessed by professional tasters	[29]
Indian black tea	A customized e-tongue setup	PCA	Taste recognizer by multi sensor e- tongue for tea quality classification	The classification of black tea liquor based on briskness, with a 85% rate	[108]
Indian black tea	Voltammetry e-tongue system, 5 noble metal-type electrodes, an Ag/AgCl reference electrode, a platinum counter electrode	PCA, LDA, BP-MLP, RBF, PNN	Much better classification ability for the combined system using the combined e-nose and e-tongue	The classification possibility of tea samples with an accuracy of 85–86% with an e-tongue	[90]
Indian black tea	Voltammetry e-tongue system, 5 noble metal-type electrodes, an Ag/AgCl reference electrode, a platinum counter electrode	PCA, FNN, BP-MLP	Tea classification using fusion of e-nose and e-tongue response using a fuzzy-based approach	FNN: the best suited model for tea classification	[68]
Indian black tea	An e-tongue with 5 noble metal-type electrodes, an Ag/AgCl reference electrode, a platinum counter electrode	Bayesian	Artificial flavor perception of tea	Improving the artificial perception when two sensory systems are fused together rather than with an individual system	[48]
Chinese green tea	Colorimetric artificial tongue, nanoporous ormosils as colorants	HCA, PCA	Discriminating nine Chinese green teas from various geographical origins and grade levels by integrating an e-nose	Efficient in characterizing compounds of high-water concentration using the developed colorimetric artificial tongue and nose system	[103]
Indian black tea	An e-tongue with 5 noble metal-type electrodes, an Ag/AgCl reference electrode, a platinum counter electrode	SVM, VVRKFA	Tea quality prediction using different types of e-tongue signal measurement	The high prediction accuracy of both the applied classifiers to assess tea quality	[109]
Black tea	An e-tongue with 5 noble metal-type electrodes, an Ag/AgCl reference electrode	FRST	A significant capability for classifying sensory properties	Better analysis of tea quality by the combined sensor response of an e-nose and e- tongue	[106]
Indian black tea	A pulse voltammetric e-tongue, 5 noble metal-type electrodes, an Ag/AgCl reference electrode	ANN, OVO-SVM, VVRKFA, PCA	Improving the classification performance of tea	Exactly predicting the tea quality among four different samples with the e-tongue signal classification	[91]
Green tea (Anji-white tea)	ASTREE II e-tongue (Alpha M.O.S., France), a reference electrode, 7 independent liquid sensors	PCA, PLS-DA	The specific geographical origins detection in Anji-white tea	High prediction sensitivity and specificity of PLSDA for e-tongue to diagnose tea taste	[101]
Longjing green tea	α-ASTREE (Alpha M.O.S. Co., France), an array of seven electrodes	KLDA, KNN	An accurate identification of tea taste and odor quality	A much better classification ability for the multi-level fusion system (e-nose + e-tongue)	[47]
Green tea	SA402B (Insent, Japan), several taste sensors array, An Ag/AgCl reference electrode	MLR, PLSR, BPNN	A theoretical reference for fast assessment of the bitter and astringent taste of green tea	The significant effect of BPNN model on the bitterness and astringency recognition of tea	[97]
Black tea	Portable e-tongue based on glassy carbon electrode and cyclic voltammetry	Si-CARS-PLS	Improving the prediction accuracy for theaflavins in tea	A fast and cheap way to measure the total theaflavins content in black tea	[89]
Longjing green teas	α-Astree (Alpha MOS Co., France), 7 liquid cross-sensitive electrodes (ZZ, BA, BB, CA, GA, HA, and JB), the Ag/AgCl reference electrode	SVM, RF, PLS	RF: the best performance in predicting the concentration of chemical components of tea	An accuracy of 100% for qualitative identification of tea quality grades, based on fusion signals by SVM and RF	[27]
Tieguanyin, Biluochun, Show bud, Westlake, and Yuzhu teas	Voltammetry e-tongue hardware system, Three-electrode module	CNN-AFE	An e-tongue for more widespread use for tea grading in the future	A ~99.9% classification accuracy for tea classification using the CNN-AFE strategy	[94]
Black tea “qi men”	Self-designed e-tongue device, 6 various cylindrical metal electrodes (outside), and a Ag/AgCl reference electrode (inside)	SRD, PLS-DA, SRD-PLS-DA	High efficiency and capability to identify the tea sample grade using e-tongue data	The potential and effectiveness of the PLS-DA-SRD model for tea grade classification	[110]
Black tea	Cyclic voltammetry e-tongue (CVET) with an glassy carbon/platinum electrode	Si-PLS, VCPA, Si-VCPA-PLS	A fast, low-cost, efficient, and complementary approach to determining free amino acids in teas	A accurate prediction of total free amino acids content in black tea using the CVET technology	[88]
5 dark teas: Fuzhuan, Pu-erh, Qingzhuan, Kangzhuan, Liubao	TS-5000Z (Insent, Japan), 6 taste sensors array [AAE, CAO, CTO, COO, AE1, and GL1]	PCA, HCA, OPLS-DA	Exploring the relationship between their taste quality (umami, sourness, saltiness, bitterness, astringency, and sweetness) and chemical profile	Negatively association between the bitterness and aftertaste-bitterness and the content of polyphenols, flavonoids, and polysaccharides of dark teas	[98]
Congou black tea	SA402B (Insent, Japan), 6 taste sensors array, an Ag/AgCl electrode	ACO, ELM, LS-SVM, PLS-DA, SVM	The taste assessment potential of tea products in the actual production process	Introducing ACO optimization algorithms for the best combination of taste features of the sensor array	[96]
Yellow tea	TS-5000Z (Insent, Japan), 5 taste sensors array	PCA, PLS-DA, HCA	The correlation determination of taste types and biochemical compositions of tea	The exact evaluation of taste properties (i.e., sweetness, umami, bitterness, astringency, and richness)	[99]
Autumn green tea	TS-5000Z (Insent, Japan), 6 lipid membrane sensors	OPLS-DA, HCA	Detecting the improved taste of tea during fermentation	The dominant taste (strong umami taste) assessment due to the presence of theabrownins	[100]
Pu-erh tea	A voltammetric e-tongue, 8 taste sensors array, the reference electrode of Ag/AgCl	CNN, BPNN, BOA	Discrimination of Pu-erh tea storage time (0–8 years)	Better Pu-erh tea identification performance by integrating an e-nose and e-tongue	[92]
Pu-erh tea	A voltammetric e-tongue, 8 taste sensors array, the reference electrode of Ag/AgCl	ELM, SVM, BPNN, CNN, TL-CNN	Discriminating the storage time of Pu-erh tea	Better pattern recognition performance of the combined deep learning and transfer learning than conventional techniques for an e-tongue	[93]
Black, White, Oolong, Green (9 samples)	A fluorescent sensor array-based e-tongue, 6 soluble conjugated polymeric nanoparticles embedded in waterborne polyurethane	LDA, SVM	Discriminating 9 tea samples with respect to tea-manufacturing	A sensing system with 100% accuracy to classify tea taste through a linear support vector machine (SVM) model	[45]

^a^ ACO: ant colony optimization, ANN: artificial neural network, BOA: Bayesian optimization algorithm, BPNN: back-propagation neural network, BP-MLP: multilayer perceptron, CNN-AFE: convolutional neural network-based auto features extraction, ELM: extreme learning machine, FNN: fuzzy neural network, FRST: fuzzy based response of signal with time, HCA: hierarchical cluster analysis, KNN: K-nearest neighbors, LDA: linear discrimination analysis, LS-SVM: least squares-support vector machine, PCA: principal component analysis, PNN: probabilistic neural network, OPLS-DA: orthonormal partial least-squares discriminant analysis, OVO-SVM: one vs. one support vector machine, PLS-DA: partial least-squares discriminant analysis, RBF: radial basis function, Si-PLS: synergy interval partial least square, Si-CARS-PLS: synergy interval partial least square with competitive adaptive reweighted sampling, SRD: sum of ranking difference, SVM: support vector machine, TL-CNN: transfer learning CNN model, VCPA: variable combination population analysis, VVRKFA: vector valued regularized kernel function approximation.

In addition, the use of various pattern discriminant algorithms has recently contributed to the recognition of associations between the tastes and biochemical profiles of tea [27,98,99]. Using a combination of non-targeted metabolomics and an e-tongue, Cheng et al. [98] found some associations between different tastes (i.e., bitterness, aftertaste-bitterness, astringency, and aftertaste-astringency) and the 49 chemical compounds of five dark teas. The results showed that the bitterness and aftertaste-bitterness decreased in the presence of polyphenols, flavonoids, and polysaccharides, whereas the content of theabrownins increased these tastes in dark teas. On the other hand, the aftertaste-astringency had a negative relationship with theabrownins, while this aftertaste was directly associated with the content of polyphenols and flavonoids. Wei et al. [99] assessed changes in chemical ingredients in yellow tea during its processing. Based on the results of PLS analysis, they found that the umami and sweetness tastes were more influenced by amino acids and sucrose, but the bitterness and astringency of yellow tea were enhanced in the presence of catechins and phenolic acids. Moreover, the red value of tea leaves was positively associated with the content of phaeophorbides. Moreover, Xu et al. [27], using a triple ESP of an e-nose, e-tongue, and e-eye, not only could qualitatively assess the quality of tea, but also efficaciously predicted the content of the main chemical compounds, including amino acids, catechins, polyphenols, and caffeine, in Longjing green teas.

### 3.3. Electronic Eye Sensors

Nowadays, the use of accurate and non-contact color measuring tools is necessary to assess the processing and quality of food products. Monitoring image information via digital detection and analysis using an e-eye instrument is possible. This technology is able to extract quantitative color data from selected areas of digital images, so that, via image processing, it can successfully distinguish inhomogeneous shapes and colors [111]. An e-eye undertakes techniques including acquisition, processing, and analysis of images. Overall, a camera takes the evaluated object’s reflected light and transfers it into electrical analog signals. After that, a computer processing system obtains the target characteristic information, selects the areas of interest, and classifies them into background and target images. The image segmentation process, which makes it possible to obtain the region of interest containing chemical information, can be performed by thresholding, edge-based segmentation, or region-based segmentation. The color information is the basis for analytical factors, whereas qualitative or quantitative analytical data can be extracted by using single calibration, pattern recognition, and multivariate analysis [27,111,112].

In various studies, e-eyes along with other ESPs (i.e., e-nose and e-tongue) have been applied to assess tea quality. Yang et al. [92] recently evaluated the simultaneous application of an e-tongue and e-eye based on deep learning, for differentiation of Pu-erh tea storage time. The applied e-eye system comprised four main parts of a five-million electronic eyepiece, the support, a light-emitting diode (LED) lamp, and an adaptor for the LED lamp. They proved that feature-level fusion, based on a deep learning algorithm, had a more accurate and robust classification performance, leading to a rapid detection, to discriminate the storage time of Pu-erh tea. Wang et al. [49] monitored withering conditions of the leaf stacks during black tea processing using a fusion of an e-eye, micro-NIRS, and colorimetric sensing array. Although the multi-technology fusion system designed in this study could not be utilized for in situ and intelligent control of the withering process of black tea leaves, the performance of this process in maintaining the quality of black tea was significantly improved.

Xu et al. [27] earlier revealed that the concurrent use of an e-nose, e-tongue, and e-eye integrated with suitable algorithms would contribute to detecting tea quality qualitatively and quantitatively. They explained that the prediction algorithms of SVM and random forest (RF) had superior performance compared to partial least squares regression (PLSR). Moreover, the regression models of RF, according to fusion signals could lead to the best prediction findings for the content of chemical ingredients. Xu and Wang [112] assessed the aroma, taste, and color signals of tea using the combined technology of an e-nose, e-tongue, and e-eye. Better performance in analyzing tea quality was obtained using fusion signals compared to the individual signals in SVM, RF, and PLSR models. In a comprehensive study, Wei et al. [99] recently monitored dynamic color changes and the significant presence of components related to umami and sweetness tastes (such as amino acids, phenolic acids, catechins, and sucrose) in tea leaves during the first 18 h of the yellowing process using an e-eye, e-tongue, and metabolomics. The yellowing process analysis after 24 h showed decreased amounts of the abovementioned compounds and increased levels of betaine, piperidine, theasinensin B, and lysophosphatidylcholines. Furthermore, the e-nose system exhibited a significant reduction in the levels of glycosidically-bound volatile compounds (i.e., benzylethyl primeveroside, linalool primeverosides, geranyl primeveroside, and linalool oxide primeverosides) in the tea yellowing step [99]. Overall, it can be concluded that the combined ESP-based technological strategy can provide an alternative system for the online management of sensory quality attributes of various food and beverage products [27,99,112].

## 4. Data Analysis and Classification Algorithms

Before the final analysis, raw data are pre-processed using a variety of methods to transform the dataset to present a better input to the PRSs, in terms of averaging, linearization, or normalization [48]. Some structural problems in the design of e-noses, such as the low performance of pumping systems, noisy recordings of gas sensors, and the remaining aroma compounds after the cleaning phase, negatively interfere with the diagnosis of the sensor array’s response to the analyte [33]. However, the determination of the best preprocessing method to improve the efficiency of the pattern recognition stage through cleaning or denoising signals is different, from one type of sensor system to another. Some of their most practical are the linear algebraic techniques of singular value decomposition (SVD), standard normal variate (SNV) transformation, background or baseline subtraction, auto scale, and baseline subtraction plus auto-scale [113]. After this necessary stage, the clustering within the datasets is mainly performed using PCA, fuzzy c-means (FCM) algorithm, and self-organizing map (SOM). Meanwhile, FCM and SOM algorithms were applied in unusual and older studies [59,114], while a PCA analysis was conducted in most ESP-based works, to cluster sensory attributes of teas with different origins, processing, and harvest and storage times (Table 1 and Table 2). The PCA not only visualizes all the information available in a dataset, but also contributes to differentiating different samples according to price, environmental, chemical, and operational variables [69]. Other unsupervised methods (such as the nonlinear mapping technique of HCA) and supervised algorithms (such as LDA, PLS-DA, SVM, and KNN) were used to classify tea samples during the monitoring of their quality using ESPs. Regression algorithms of PLS and PLSR (linear), artificial neural networks (ANNs; such as MLP, LVQ, PNN, and RBF (nonlinear)), and support vector regression (SVR, nonlinear) in combination with ESPs could well classify and simplify the data of the sensory properties of tea. Furthermore, using a back-propagation neural network (BPNN) and RF models as supervised learning algorithms showed a good ability to lessen the mean square error between the computed and desired outputs of the network, to discriminate teas with dissimilar attributes (Table 1 and Table 2).

**Figure 5:**
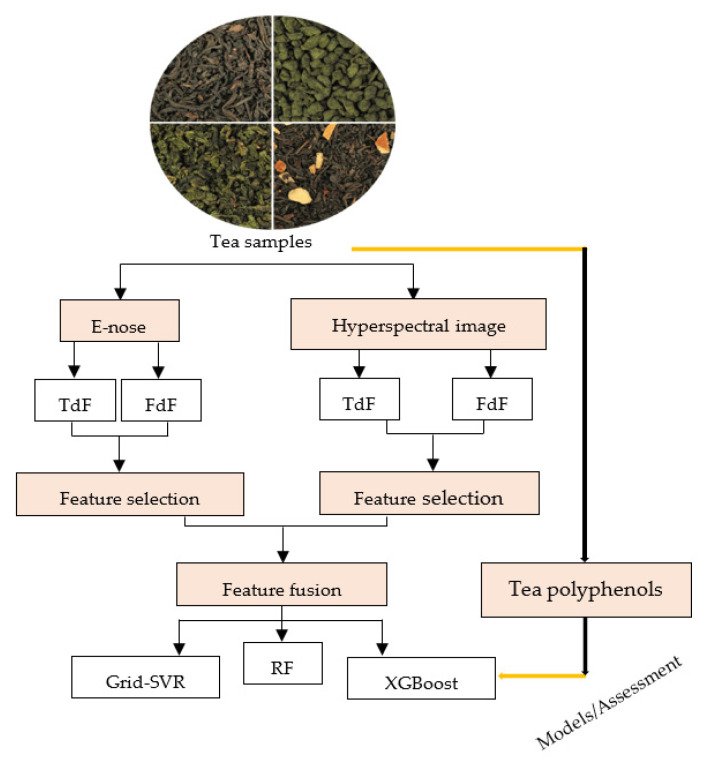
A diagram of a polyphenol evaluation model present in black, green, and yellow teas, based on feature fusion of an e-nose and hyperspectral imagery. FdF: frequency-domain feature, TdF: time-domain feature, Grid-SVR: grid support vector regression, RF: random forest, XGBoost: extreme gradient boosting. Retrieved from Yang et al. [77].

The quality of food materials such as tea can be quantitatively determined using proper algorithms, by discovering the correlations between ESP signals and quality indices. On the one hand, a high number of chemical compounds are involved in the sensory quality of tea; while, on the other, most of these ingredients are related to each other in producing the aroma, taste, and color quality of tea. Thus, the fusion signals of ESPs might be efficient in assessing the content of chemical compounds in tea types [46,112]. Since the main part of human perception is a consequence of multiple sensory organs, data fusion can deliver more accurate information about organoleptic characteristics compared to a single ESP. In general, the fusion is classified into three groups: low-level (data fusion, DaF), intermediate-level (feature fusion, FeF), and high-level (decision fusion, DnF). The DaF integrates the raw sensor response signals into a sole signal, whereas the features extracted from the sensor signal amounts in FeF are interconnected with a diversity of feature extraction and selection techniques. Finally, DnF, by combining decisions from several sensory channels, exhibits the output of multiple classifiers, to attain an ending forecast, which is similar to the fusion structure in the human brain. To sum up, fusion contributes to a better description and more accurate classification of data by combining the information obtained from multiple sources [47,115]. The best classification models for discriminating tea quality grades based on fusion signals with an accuracy of over 90% were SVM and RF [27], K-nearest neighbors (KNN) [47], SVM [44,45,96], extreme gradient boosting (XGBoost) [77], kernel-based LDA (KLDA)-KNN [69], fuzzy neural network (FNN) [68], the Bayesian approach [48], and PCA and KNN (71). Moreover, Yang et al. [77] recently determined the feasibility of developing a cross-category model for predicting tea polyphenols based on the feature fusion of an e-nose and hyperspectral imagery (HIS). They confirmed that the accuracy of the fusion features, according to frequency-domain feature (FdF) and time-domain feature (TdF), obtained from the e-nose and HIS systems was much more than the features from each sensor. Among the three regression models of RF, XGBoost, and Grid-SVR, the efficiency of XGBoost as a decision-tree-based ensemble machine learning algorithm, compared to the two other models, was higher due to the increase of estimation accuracy for tea polyphenol content (Figure 5).

## 5. Conclusions and Future Trends

This overview showed that the use of ESPs as non-destructive, and automated instruments not only accelerates the online monitoring of tea quality but also significantly reduces the assessment time and cost for the specialists and personnel of tea factories globally. The use of these intelligent devices is an efficient alternative for testing experts, to determine tea quality through the organoleptic evaluation of aroma, taste/aftertaste, color, and mouthfeel scores. In general, ESPs were used for the qualitative and quantitative classification of tea samples, with the same/various geographical origins, grades, as well as harvest and storage times. The analysis of output data from each electronic sensor system using machine learning algorithms and statistical prediction models could adequately classify tea based on the target quality properties. The optimal prediction model for tea quality discrimination was varied, because diverse feature selection algorithms were examined on dissimilar datasets. Furthermore, the combination of signals obtained from two or three ESPs (e-nose, e-tongue, and e-eye) with a feature-level fusion strategy could significantly enhance the efficiency of identification and prediction models. This quality classification led to the recognition of biochemical compounds and their relationships with the profile of taste, color, and aroma changes during storage and processing.

According to the promising results of using ESPs in maintaining and improving the quality of tea, similar e-sensors integrated with superior feature representing methods can be used to predict and classify the sensory quality of other food products (such as fruit juices, soft drinks, plant and animal milk, and vegetable oils), for quickly satisfying the demands of both consumers and the market. There has been tremendous growth in presenting machine learning methodologies in classifying tea quality. It is expected that the new, efficient frameworks can extract deep features from various sensor signals, to perform tea authentication with more sensitivity and accuracy in the future. Meanwhile, the capability of diverse kinds of features can be assessed using various signal transformation methods. Since the combination of triple ESPs has been performed less frequently, the simultaneous application of an e-nose, e-tongue, and e-eye may result in all-around data support for a wide-ranging quality assessment of tea and other foods/beverages. Moreover, it is interesting to compare the results obtained from ESPs, (FT-)NIRS, HPLC-diode-array detection (DAD) fingerprints coupled with chemometric analysis, etc., to correctly evaluate the performance of e-sensors in the quality control of tea samples. A serious effort should be made to design and develop advanced micro- and nano-e-sensors, followed by their comparison with smartphone-based micro-NIRS. Another important issue is the production of self-adhesive bioelectrodes in the structure of ESPs, for the stable detection of sensory attributes under harsh environmental conditions, so that they can sensitively simulate human physiological signals for food quality assessment. Last, it is recommended to compare the online variation in healthy-functional compounds and health benefits of tea using single and combined EPSs in the future.

## Data Availability

Not applicable.
